# Peer observation of teaching as a faculty development tool

**DOI:** 10.1186/1472-6920-12-26

**Published:** 2012-05-04

**Authors:** Peter B Sullivan, Alexandra Buckle, Gregg Nicky, Sarah H Atkinson

**Affiliations:** 1 Department of Paediatrics, Children’s Hospital, University of Oxford, Oxford, OX3 9DU, UK

**Keywords:** Peer observation, Feedback, Medical education

## Abstract

**Background:**

Peer observation of Teaching involves observers providing descriptive feedback to their peers on learning and teaching practice as a means to improve quality of teaching. This study employed and assessed peer observation as a constructive, developmental process for members of a Pediatric Teaching Faculty.

**Methods:**

This study describes how peer observation was implemented as part of a teaching faculty development program and how it was perceived by teachers. The PoT process was divided into 4 stages: pre-observation meeting, observation, post-observation feedback and reflection. Particular care was taken to ensure that teachers understood that the observation and feedback was a developmental and not an evaluative process. Twenty teachers had their teaching peer observed by trained Faculty members and gave an e-mail ‘sound-bite’ of their perceptions of the process. Teaching activities included lectures, problem-based learning, small group teaching, case-based teaching and ward-based teaching sessions.

**Results:**

Teachers were given detailed verbal and written feedback based on the observer’s and students’ observations. Teachers’ perceptions were that PoT was useful and relevant to their teaching practice. Teachers valued receiving feedback and viewed PoT as an opportunity for insight and reflection. The process of PoT was viewed as non-threatening and teachers thought that PoT enhanced the quality of their teaching, promoted professional development and was critical for Faculty development.

**Conclusions:**

This study demonstrated that PoT can be used in a constructive way to improve course content and delivery, to support and encourage medical teachers, and to reinforce good teaching.

## Background

The General Medical Council which regulates medical practice in the United Kingdom has, in its 2009 report ‘Tomorrow’s Doctors’, set the standards that it will use to judge the quality of undergraduate teaching and assessments in individual medical schools. Two quotations from this report give indications of this which are relevant to the present paper:

"‘Everyone involved in educating medical students will be appropriately selected, trained, supported and appraised’"

"‘The medical school must ensure that appropriate training is provided…and that staff development programmes promote teaching and assessment skills’"

The aim of this study was to address both of these issues within the context of an undergraduate pediatric course. As part of an ongoing process of course and faculty development a peer observation of teaching (PoT) process was offered as a developmental opportunity for members of the teaching Faculty.

PoT involves observers providing descriptive feedback to their peers on learning and teaching practice [1] and can be seen as a means by which the quality of teaching and learning process in higher education establishments is both accounted for and improved [2]. PoT has attracted increasing attention in higher education in recent years. This arises, in part, to help prepare for internal or external audit of teaching as, for instance, in HEFCE-driven assessments of university teaching and is also partly a reflection of the awareness of the need to foster teacher development and professional growth and to adapt to the changing demands of the higher education system [3].

A consequence of these two drivers is the potential for confusion or conflict about the role of the observer. On the one hand, with evaluation and audit-driven process there is the possibility that observation may acquire a threatening, confrontational dimension, which may alienate the teacher. On the other hand, and probably depending in large measure on how it is approached, the peer observation process may be perceived by the teacher as a constructive, developmental adjunct to their teaching, which improves opportunities for student learning.

In view of this possible controversy, there is a need for clear focus and goals: ‘we should be very clear about exactly what our objectives are for the implementation of peer observation, and the best way to achieve these, before espousing a potentially divisive and detrimental procedure’ [3]. Shortland states that ‘an inappropriate choice of methodology – may lead to de-motivating feedback, presenting a dilemma within observation practice’ [1]. This is obviously a major concern and one that is not only represented in the literature, but in actual practice. At its worst, the aims of this exercise introduce ‘conflict’ in a system that is meant to inspire ‘confidence, enthusiasm and a sense of professional worth’ [3]. As one case report states: ‘Peer observation was designed to meet the twin aims of teacher development and quality assurance. Teachers’ views suggest these two aims may conflict’ [4].

Ramsden points out that ‘there can be no single right answer to the problem of improving the quality of university teaching’ [5]. If peer observation feedback is to achieve its goal of being motivating and helping people to learn [5], then it must be remembered that it is not an ‘automatic recipe for enhanced learning and development’ [1]. However, research unequivocally indicates that ‘classroom observation methodologies…can provide a different perspective on the observation process and thus play a part in developing observers as reflective practitioners of teaching and learning’ [1]. Irrespective of the reason for observation of teaching, it is imperative that the process is conducted in a structured and managed fashion. As Fullerton observes, ‘The aim of the observation is to help improve the skills of the observed, therefore quality feedback is essential’ [6].

Despite a large literature on PoT, there are few accounts of its implementation in clinical teaching [7] and as far as we are aware no accounts of clinical teachers’ perceptions of PoT. The aims of this project were firstly to implement PoT methods as a constructive, developmental process for members of the Pediatric Teaching Faculty and secondly to assess teachers’ perceptions of the PoT process. Our overall aim was to improve opportunities for student learning in pediatrics in our institution.

## Methods

Peer observation was undertaken by Faculty members (PBS and SHA) with specific training in PoT provided by a Fellow of the Higher Education Academy with specialist knowledge of PoT. There was one-to-one training in the techniques involved followed by peer observation of trainee observers’ teaching. Our 8 week pediatric course is presented 6 times a year with the ensuing danger of becoming mechanical and stale. We therefore assessed that there was a need for PoT to keep our course material and lectures up to date and to affirm the efforts of our teaching Faculty. Critically, and this was emphasized to teaching Faculty, the PoT process was developed to be constructive and developmental. As discussed in the literature [1], an inappropriate methodology might lead to de-motivating feedback and would not achieve our aim of improving student learning.

Between October 2008 and January 2011, 15 Consultants (by PBS), 3 Clinical Lecturers and 2 Specialist Registrars (by SHA) were peer observed. Only 4 Consultants regularly contributing to the undergraduate course declined the invitation. The reasons given for declining were: from the most senior (n = 2) “not necessary”; from the most junior “too busy” and the fourth misunderstood the process and has subsequently agreed to participate. The teaching activities observed included 10 lectures, 2 problem-based teaching sessions, 3 small group teaching activities, 3 case-based teaching sessions and 2 ward-based teaching rounds. The teaching sessions were generally about one hour long. The pre-observation meeting generally took between 15 and 20 minutes and the post-observation feedback about 25–30 minutes. Each observation therefore took about 2 hours.

**Figure 1:**
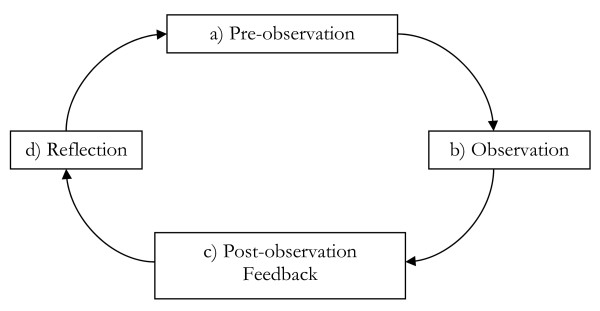
**Peer Observation Process (Bell 2002,**[8]**]).**

The general approach that was adopted for peer observation of teaching was based on Bell’s model [8]. Figure 1 illustrates the cyclical nature of the process.

This approach will now be discussed under these four sub-headings:

· Pre-observation meeting

· Observation

· Post observation feedback

· Reflection

### Pre-observation meeting

Prior to the observations, a pre-observation meeting was held to clarify the process and enquire of the teacher what they required from the review and to establish the context of the teaching event. Topics covered at this meeting were;

· Context of the teaching; how the session fits into the course

· The content and its place within the curriculum of the unit and the programme of study

· To what extent is this session relied upon to deliver teaching on the whole topic

· Identify specific learning objectives for this session

· Teaching approach to be adopted, anticipated student activities, time plan for the session

· Any potential difficulties or areas of concern

· Any particular aspects that the tutor wishes to have observed

· How the observation is to be conducted

· The way in which the students will be informed and incorporated into the observation

· Any particular concerns that either the observer or the observed might have about undertaking the observation.

This pre-observation meeting is an essential component in establishing a ‘contract’ with the teacher to underline that this is intended to be a developmental exercise and not an evaluative/assessment process [9].

### Observation

During the observation notes were taken on the content, style and delivery of the teaching and these were used to inform the post observation feedback. With the teacher’s approval a short questionnaire scored with a Likert scale and with space for free text comments (Additional file 1: Appendix 1) was administered to the students at the end of each observed session. The purpose of this was to help validate any observation made by the observer.

### Post observation feedback

The model of feedback for each peer observation was broadly based on the revised Pendleton rules [10]. The purpose of giving feedback has been well summarized by King (1999), ‘Giving feedback is not just to provide a judgment or evaluation… It is to provide insight’ [11]. If feedback is to be effective certain criteria must be met. Feedback should be:

· Descriptive - of the behavior rather than the personality

· Specific - rather than general

· Sensitive - to the needs of the receiver as well as the giver

· Directed - towards behavior that can be changed

· Timely - given as close to the event as possible

· Selective - addressing one or two key issues rather than too many at once

At the end of the feedback session the observer and the observee examined and discussed the results of the student questionnaire. Potential solutions to any concerns raised were collaboratively identified and discussed by the observer and observee. Each teacher received a letter providing a written summary of the outcome of the observation process assimilating both the observer’s comments and the students’ comments together with potential solutions to any concerns raised.

### Reflection

An important component of peer observation is the opportunity for teachers to reflect on their teaching in the light of feedback from observation. All participants were invited to reflect on their observation and to send an email with comments on their experience of the process and what, if any, value it had for them as teachers. This informal approach was considered to be more likely to achieve a response rather than any structured or formal approach such as using a questionnaire.

### Data analysis

Reflective feedback from the teaching faculty on PoT was analyzed using qualitative methods. Key themes in the data were identified and content analysis was carried out via systematic coding using NVivo Version 9 (QSR International Pty Ltd, Doncaster, Australia). Data were analyzed using a grounded theory approach [12] with constant comparison. The use of direct quotation gave additional richer perspectives on how, when and why certain observations were made [13].

## Results

### Post-PoT recommendations

Observation of teaching activities provided an opportunity to examine both content and delivery of individual course components so that suggestions could be made as to how these might be improved or refined. Some examples of these post-PoT recommendations to individual teachers are listed: 

· Ensure that learning objectives for the session are defined

· Refinement of slides by updating old slides and removing unnecessary ones

· Embed video clips in PowerPoint rather than switching to VHS format mid-lecture

· Convert Video to DVD to prevent further deterioration of useful teaching material

· Improve interaction with students

· Update teaching materials on course website e.g. use up to date growth charts

· Avoid “contamination” in small group sessions too close together in a small room

· Improve session structure with less jumping backwards and forwards between topics

· Identify what adult medicine teaches (e.g. Diabetic Ketoacidosis) and ensure consistency

The following letter extracts give a sense of how suggestions for improvement were handled:

"‘One of the disadvantages, of course, of using the white board is that one can end up talking to the white board with one’s back to the students’"

"‘I thought a couple of slides which you used could be ditched and we discussed that in our post-observation de-brief. I think this will help deal with some of the time pressures that you were experiencing’"

The Peer Observation process was also useful to reinforce good teaching as the following letter extracts demonstrate:

"‘The presentation was very lively and interactive and well illustrated with case studies. I particularly liked your stick diagram to illustrate the differential diagnosis of Wilms’ tumor and neuroblastoma’"

### What about the observees?: the reflective component of PoT

The device of using an email ‘sound-bite’ to document evidence of the reflective component of Peer Observation was vindicated by the 100% response rate from observees. Seven major themes emerged from the data. These were: usefulness and relevance; value of feedback, insight and reflection; non-threatening process; enhanced teaching quality; professional development; and the necessity of peer observation for Faculty development.

### Usefulness and relevance

PoT was overwhelmingly described by the Teaching Faculty as extremely useful, valuable and relevant to their teaching practice.

"‘I actually thought that the whole process was extremely useful and relevant’"

### Value of feedback

A major theme was that of the value of feedback. Teachers strongly valued receiving feedback from the observer and from students and thought that it improved their performance. An important component of this was receiving ‘immediate feedback’.

"‘One very rarely gets feedback – positive or negative on teaching so it was an interesting and worthwhile experience’"

"‘Live feedback can only improve one’s teaching overall’"

"‘Useful to have feedback from the perspective of both the students and another teacher’"

### Promotion of insight and reflection

Another major theme was that PoT gave teachers insight and promoted reflection on their teaching practice.

"‘Peer review is an essential way of gaining a perspective on one’s teaching’"

"‘It made me look critically at the presentation…think more clearly about my objectives’"

"‘All too often teaching takes place without the opportunity for this kind of reflection’."

### Non-threatening process of PoT

The overwhelming majority of teachers thought that the process of PoT was constructive and non-threatening, although the potential for the process to be threatening was acknowledged. The peer aspect of the process was also appreciated.

"‘Helpful and non-threatening feedback on teaching skills’"

"‘The way in which the observation was conducted was considerate and unobtrusive’"

"‘Less threatening than a more ‘senior’ member of the teaching faculty sitting in on a session’"

"‘When done in a sympathetic, but informed way, this is a helpful tool’"

### Enhanced teaching quality

Teachers described the tangible improvement in their teaching practice that had resulted from the detailed and specific feedback they had received from PoT. The overwhelming perception of the teachers was that these changes had resulted in enhanced quality of learning for the students.

"‘I was able to make some useful changes to the lecture that has already led to improvements in the session’"

"‘Forced me into improving my audio-visual aids…which I had been meaning to do’"

"‘Resulted in a more effective teaching experience for the students’"

### Professional development and worth

Teachers thought that PoT enhanced their professional development and feelings of worth. ‘I was fairly confident that students liked my presentations and that it was a fairly interactive session, but hearing from them and you formally just boosted that belief and confidence’

### A necessary and important process

Finally, PoT was described by teachers as a necessary and important process in a Teaching Program. The teachers advocated that PoT should be more widely implemented.

"‘If we do not do this we are at risk of doing the same old thing without variation. I am sure that there are some academics who give the same talk today as 20 years ago – is this the way ahead? I think not. If you are not open to learning then you should not teach’"

"‘I would recommend peer review to all teachers…should be used more widely’"

### Benefit for the observers

The process of training to be an observer and implementing peer observation was also of benefit to the observers’ professional development. It promoted awareness and reflection on one’s own teaching style and content and it was useful to learn from and borrow teaching techniques from other teachers.

## Discussion

This study has shown that PoT can be used as a technique both to update and refine the content and delivery of a well-established teaching course, and to provide useful feedback to teaching Faculty. This technique is useful therefore, to Course Directors who rarely get on opportunity to see the fine detail of the content of course materials or to witness the interaction of teaching faculty and students in the front line. As a result of frequent repetition (our 8 week course is presented 6 times each year) it is easy for lectures to become stale and mechanical. Power Point-based lectures may be inherited from previous teachers and/or repeated from course to course and from year to year without being updated as new information arises. An example of this last point was the use of the 1990 Growth Charts for children rather than the World Health Organization growth charts in widespread use since 2009 in a teaching module on Normal Growth and Development. Introduction of an impartial but informed observer into the teaching session has been shown to be a relatively straightforward way of keeping the course material up to date and refreshing and reaffirming the teaching style of the lecturers. An important part of the process is ‘building a partnership’ or ‘working alliance’ between the observer and observee [14], and giving specific feedback that is focused on the task and in line with personal goals [15]. In agreement with a study on the implementation of PoT in pharmaceutical education we found that a particular strength of the process was the pre-observation meeting which allowed for ‘customization of the process to meet the Faculty member’s specific needs’ [16].

Teaching Faculty unanimously described the PoT process as very useful and relevant to their teaching practice and teachers appreciated the opportunity to discuss their teaching and to have constructive feedback. The success of this process was in no small measure related to the efforts expended on emphasizing that it was not an evaluative assessment but being applied by an equal as a professional developmental tool. There is little doubt that when used in such a positive way peer observation encourages and supports teaching Faculty. However, as a GP questionnaire revealed, anxiety is likely to be provoked if PoT is imposed from outside and is not conducted by a peer [4]. Moreover, as noted in another study, PoT also gave the observing teachers the opportunity to reflect on their own teaching practice and to borrow effective teaching techniques [7].

This study has also shown how important it is to individual lecturers to receive immediate feedback from students. At the end of each course students are required to complete the Oxford Course Evaluation Questionnaire which is used to assess the students’ perceptions about teaching, workload, goals, standards and assessment methods [17]. It is based on this ongoing evaluation that we know that the course is successful in achieving its stated aims and objectives and that the great majority of students are satisfied with the organization and delivery of the course. Nevertheless, only occasionally do individual teachers get singled out for special mention so the immediate feedback provided by the simple questionnaire designed for this study enabled lecturers to see how their own lecture was received by the students. Not all comments from students were positive. Examples included ‘spoke too quickly’, ‘too many slides’, ‘rushed at the end’, but when used in conjunction with the feedback from the observation these comments had a confirmatory effect and were taken constructively by the lecturers.

The advantages of PoT when adopted in this developmental way are clear. Teachers described tangible improvements in the quality of their teaching and an enhancement of their professional development and worth. Nevertheless, it is important to emphasize the limitations of PoT. The successful application of PoT requires expertise, time and commitment. The fact that it took 30 months to complete 20 observations (at 2 hours each) indicates that the time factor is a significant limitation. This is in agreement with another study which has emphasized concern regarding ‘the time it will add to an already heavy workload’ [16]. In future it is intended that PoT will be offered to all new lecturers and re-offered to existing lecturers either on request or every five years. It is also hoped that other Faculty members may be willing to acquire the skills necessary to undertake PoT and so share the workload.

This study had a number of limitations. The department of Pediatrics is relatively small and only three peer observers have been trained to date although there are plans for more Faculty members to be trained in this process. There was also a challenge with other time pressures to complete the post-observation meeting and letter in a timely fashion. However, we believe that giving immediate feedback is one of the most important aspects of the process and consequently prioritized the post-observation feedback. Another potential limitation of the study was the lack of anonymity with the e-mail ‘sound-bite’ received from the teachers. We do not think that this is likely to have influenced our results as feedback revealed that teachers felt very comfortable with the peer aspect of PoT and did not view the process as threatening.

## Conclusions

In summary, our study showed that PoT can be effectively implemented within an undergraduate pediatric curriculum for the development of the teaching staff and ultimately to improve the quality of student teaching.

## Practice points

Peer Observation of Teaching can be used to:

· identify the need to update teaching course materials

· demonstrate to students departmental commitment to good teaching practice

· reaffirm good teaching skills of teaching faculty

· provide developmental feedback to help faculty refine teaching methods

· maintain high standards in undergraduate teaching

## Competing interests

The authors declare that they have no competing interests.

## Authors’ contributions

PS is Director of Teaching, Learning and Assessment and Head of the University of Oxford, Department of Paediatrics. He is a qualified Physician Educator of the Royal College of Physicians of London and a Fellow of the Higher Education Academy. He conceived and carried out the study and wrote the paper. AB is tutor at the University of Oxford and holds the University’s Postgraduate Diploma in Learning and Teaching in Higher Education and is a Fellow of the Higher Education Academy. She has a special interest in Peer Observation and advised on the design of the study and contributed to writing the manuscript. NG is Course Administrator for the undergraduate paediatric course in the University of Oxford, Department of Paediatrics and was responsible for the logistic and administrative arrangements of the study. SA is a Clinical Lecturer in the Department of Paediatrics, University of Oxford with a special interest in Medical Student Education. She contributed to carrying out the study, analysis of data and writing of the manuscript. All authors read and approved the final manuscript.

## Pre-publication history

The pre-publication history for this paper can be accessed here:

http://www.biomedcentral.com/1472-6920/12/26/prepub

## Supplementary Material

Additional file 1: Appendix 1. Feedback form. Click here for file
