# Supplementary material for: Peer observation of teaching as a faculty development tool
Source: BMC Med Educ. 2012 May 4;12:26. doi: 10.1186/1472-6920-12-26 (PMC3406982; doi:10.1186/1472-6920-12-26)
Supplement: Additional file 1: Appendix 1. — Feedback form. [file 1472-6920-12-26-S1.doc]

APPENDIX 1

**Feedback form**

**Lecturer**: **Date**:

Title:

|  | Circle appropriate number | | | **Comments** |
| --- | --- | --- | --- | --- |
| VOICE | Clearly audible; well mounted | 5 4 3 2 1 | Largely inaudible; monotone |  |
| PACE | Appropriate number of words and ideas in the time | 5 4 3 2 1 | Too fast or too slow; too many or too few ideas |  |
| NON-VERBAL  COMMUNICATION | Good use of gestures and eye contact; no distracting mannerisms | 5 4 3 2 1 | Poor or distracting use of gestures and eyes; annoying mannerisms |  |
| ORGANISATION AND PREPARATION | Well organised, e.g., introduction to topic, planned repetition, summaries, links between ideas well explained | 5 4 3 2 1 | Badly organised; confusing presentation; poorly explained |  |
| USE OF OHP, AUDIOVISUAL AIDS ETC. | Clear; well presented  Appropriate use; supportive to presentation | 5 4 3 2 1 | Cramped, illegible; inappropriate use; detracts from presentation |  |
| ATTITUDE | Friendly, responsive, enthusiastic; creates positive impression | 5 4 3 2 1 | Creates negative impression; dull, unfriendly, unresponsive |  |
